# Six new subterranean freshwater gastropod species from northern Albania and some new records from Albania and Kosovo (Mollusca, Gastropoda, Moitessieriidae and Hydrobiidae)

**DOI:** 10.3897/subtbiol.23.14930

**Published:** 2017-10-26

**Authors:** Jozef Grego, Peter Glöer, Zoltán Péter Erőss, Zoltán Fehér

**Affiliations:** 1 Horná Mičiná, SK-97401 Banská Bystrica, Slovakia; 2 Biodiversity Research Laboratory, Schulstr. 3, D-25491 Hetlingen, Germany; 3 Department of Zoology, Hungarian Natural History Museum, H-1083, Baross utca 13, Budapest, Hungary; 4 3rd Zoological Department, Natural History Museum, Vienna, Burgring 7, A-1010, Vienna, Austria

**Keywords:** cave, spring, Kosovo, *Paladilhiopsis*, *Iglica*, *Plagigeyeria*, *Saxurinator*, stygobiont, interstitial water

## Abstract

During a field trip to the western part of the Balkan Peninsula in 2016, investigations of several caves and karstic springs revealed six new gastropod species living in subterranean waters and resulted in some note-worthy faunistic records. Five of the new species are assigned to the genus *Paladilhiopsis* Pavlović, 1913, namely *P. prekalensis* **sp. n.**, *P. lozeki* **sp. n.**, *P. szekeresi* **sp. n.**, *P. wohlberedti* **sp. n.**, *P. falniowskii* **sp. n.** and one to the genus *Plagigeyeria* Tomlin, 1930, namely *P. steffeki* **sp. n.** New Albania and Kosovo distribution records are given for *Iglica illyrica* Schütt, 1975, *Plagigeyeria zetaprotogona* Schütt, 1960, *Vinodolia matjasici* (Bole, 1961), and the first georeferenced record is given for *Saxurinator schlickumi* Schütt, 1960. The most important environmental factors influencing habitat selection of these subterranean freshwater gastropods are briefly discussed.

## Introduction

It is known that the Balkan Peninsula is one of the most significant hotspots in terms of subterranean freshwater gastropod diversity (Radoman 1983, Bole and Velkovrh 1986; Kabat and Hershler 1993; Strong et al. 2008). In light of this, the number of the species known from Albania is surprisingly low, which might be due to the fact that the Albanian fauna is still very poorly explored compared to the other parts of Balkan Peninsula. The majority of hitherto known subterranean gastropod records is concentrated to the Lake Skadar area (also known as Lake Scutari or Lake Shkodër) with only a few additional records from the northeastern and southern parts of Albania (Reischütz and Reischütz 2008; Erőss and Fehér 2009; Pešić and Glöer 2013a; Glöer et al. 2015; Reischütz et al. 2013, 2014, 2016). Live collected specimens remain very scarce, therefore, most of the studies are based only on shell morphology. Likewise, all of the descriptions in this study based exclusively on shell characters, and therefore, these genus assignments remain somewhat uncertain and provisional until they are confirmed by anatomical and molecular data. In addition to the new subterranean freshwater gastropod taxa, our investigations of spring and cave localities in Albania and Kosovo also resulted in interesting novel distributional data. Due to the hitherto scarce records of all subterranean species from Albania, they significantly contribute to a better understanding of their zoogeography and distributional pattern.

## Material and methods

**Figure 1:**
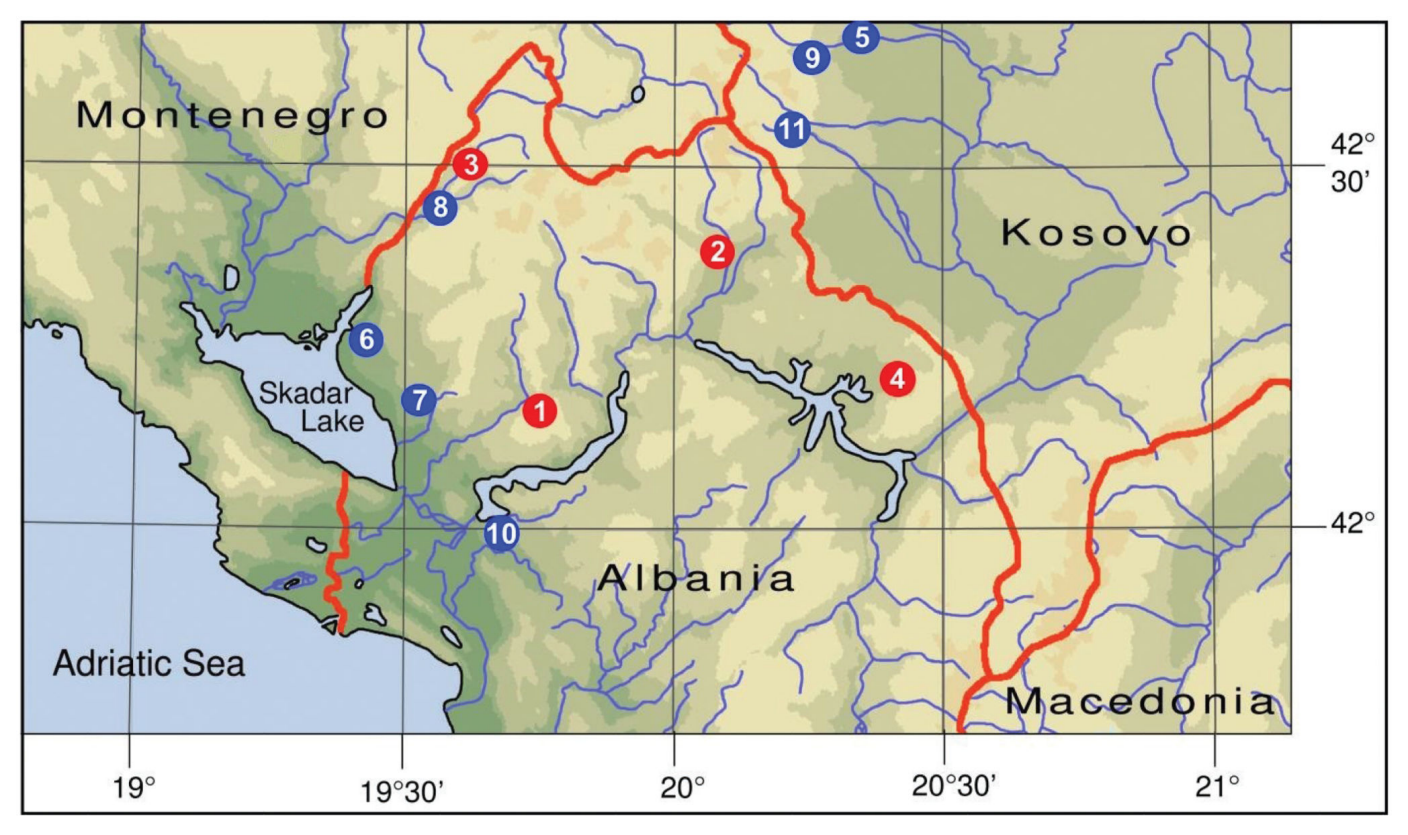
Distribution of the new species and faunal records mentioned in this paper. Red dots indicate type localities of new the species. **1** Prekal, Zhyla Cave (*Paladilhiopsis prekalensis* sp. n.) **2** Shoshan (*Paladilhiopsis lozeki* sp. n.) **3** Tamarë (*Paladilhiopsis szekeresi* sp. n. and *P. wohlberedti* sp. n.) **4** Krumë, Vrela Spring (*Paladilhiopsis falniowskii* sp. n., *Plagigeyeria steffeki* sp. n. and *Paladilhiopsis szarowskae*) **5** Novosellë, White Drin Spring (type locality of *Plagigeyeria gladilini* and *Iglica illyrica*) **6** Bajzë, Syri i Sheganit Spring (*Plagigeyeria zetaprotogona*) **7** Rrash, Vrak Spring (*Plagigeyeria zetaprotogona*) **8** Tamarë, Dverpte Cave (*Plagigeyeria zetaprotogona*) **9** Kosovo, Pejë, Rugova Gorge, (*Saxurinator schlickumi*) **10** Vau i Dejës (type locality of *Paladilhiopsis gittenbergeri*) **11** Kosovo, Decani Valley (*Iglica illyrica*).

**Figure 2:**
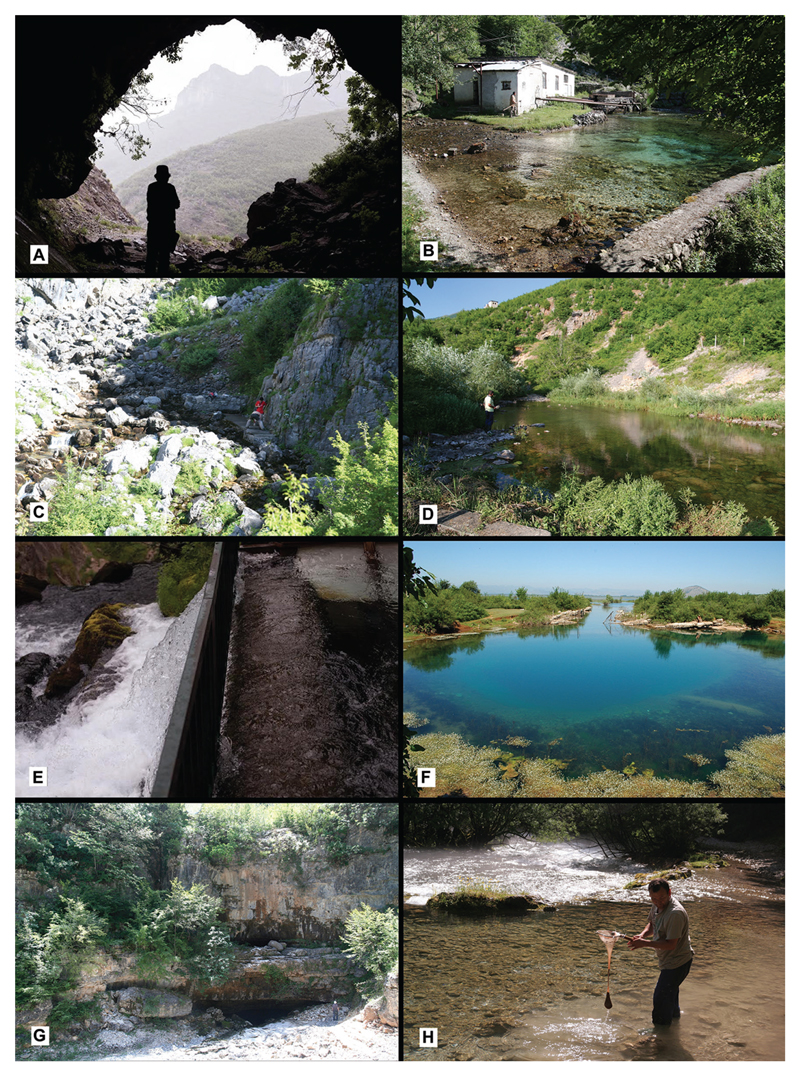
Various habitats where subterranean gastropods were found: **A** Prekal, Zhyla Cave **B** Shoshan Spring **C** Tamarë, spring above the trout farm **D** Krumë, Vrela Spring **E** Novosellë, White Drin Spring **F** Bajzë, Syri i Sheganit Spring **G** Dverpte Cave south of Tamarë **H** sampling from sand and fine gravel by a sieve combined with polyamide pantyhose (photos: Zoltán Fehér, Maroš Grego, Dávid Murányi).

The studied material was collected during a field trip in June and July of 2016 in northern Albania and Kosovo (Fig. 1). Various cave outflows and karstic springs were studied, where we preferred the following micro-habitats for sampling: (i) sedimentation zones of springs immediately at (or close to) the spring outlet zone, (ii) calm sedimentary spots of cave streams and (iii) from the scarce fine sand and gravel trapped among larger stones in higher water velocity sections of cave streams. In the case of dried-out intermittent springs and cave rivulets the dry sand sediments from the bed had been sampled in the same way. Sampling was carried out by a metal sieve of 3 mm mesh size combined with a polyamide knee-sock or pantyhose of ca 0.2 mm mesh size. Sand and fine gravel were sifted through the sieve. The material retained in the hosiery was further washed *in situ* by fresh water to remove the muddy and the very fine sand fractions of the sediment (Fig. 2H). We have used new hosiery at each sampling site. About 0.05–0.5 kg samples were collected per locality and immediately fixed in ca. 75% ethanol. Samples were sorted under Olympus SZ-11 stereo microscope. In the first round, the samples were sorted wet and if shells containing soft parts were found, they were transferred into fresh 75% ethanol with 20% ambient amount against the estimated specimen volume. After this, the samples were then dried and sorted through again to gain a second crop of dry shells which might have been overlooked during the wet sorting. Frontal, ventral and lateral view images were made by a digital camera system (Leica R8, Leitz Photar 21mm objective with Novoflex bellows and by Olympus DP-10 camera) and ImageJ scientific image analyzing software was used for making the measurements together with direct measurement with eye-piece micrometer.

## Results

The shells of the genus *Bythiospeum* and *Paladilhiopsis* are very similar and it is not possible to distinguish between these genera by shells only. An ongoing molecular study of the *Moitessieriidae* Bourguignat, 1863 following the earlier studies of Szarowska (2006) could not confirm the presence of genus *Bythiospeum* Bourguignat, 1892 in the Balkans, while the genus *Paladilhiopsis* Pavlović, 1913 was proved to be very diverse over the studied territory (Hofman et al, in MS). Thus, we place all the species described here as new with the shell shape like *Bythiospeum*/*Paladilhiopsis* to the genus *Paladilhiopsis*. For the same reason the *Bythiospeum szarowskae* Glöer, Grego, Erőss & Fehér, 2015 is treated as *Paladilhiopsis szarowskae*. In addition, a molecular phylogenetic reconstruction using the nuclear Histone H3 marker revealed that *Bythiospeum blihensis* Glöer & Grego, 2015 as well as *Iglica gittenbergeri* Reischütz and Reischütz, 2008 belong to the genus *Paladilhiopsis* (Andrzej Falniowski, personal communication) as well. The *Plagigeyeria steffeki* sp. n. shell morphology is well aligned with the related species hitherto assigned to that genus.

**Superfamily Truncatelloidea J.E.Gray, 1840**

**Family Moitessieriidae Bourguignat, 1863**

**Genus *Paladilhiopsis*Pavlović, 1913**

***Paladilhiopsis prekalensis* sp. n.**

http://zoobank.org/59D6C1B8-7543-4ED9-AAB2-CAF2D17A443B

Figures 3–4

**Diagnosis.** Can be compared to *Paladilhiopsis szekeresi* sp. n. (Albania, Tamarë), from which it differs by a blunter apex, more convex whorls, more elongated and more declined aperture from the columella and by upper aperture edge more prominent in the shell outline as well as by the narrower umbilicus. It differs from *Paladilhiopsis lozeki* sp. n. (Albania, Shoshan) by its smaller and less elongated shell, blunter apex, more tumid body whorl and by the shape of the aperture. *P. wohlberedti* sp. n. (Albania, Tamarë) and *Montenegrospeum bogici* Pešić & Glöer, 2012 (Montenegro, Podgorica) differ markedly by their larger more conical shells, more prominent body whorls and shape of their apertures. The shell shape of the news species is somewhat similar to that of *Bosnidilhia vreloana* Boeters, Glöer & Pešić, 2013 (Bosnia, Banja Luka) which differs from the new species by its more slender shape, narrower elongated aperture and more flattened whorls.

**Type locality.** Albania, Shkodër district, Prekal, Shpellë e Zhylit (Zhyla Cave), 210 m, near junction of Kiri and Prroni i Cise, 205 m, 42.1782°N; 19.7200°E (Fig. 2A).

**Type material.** Holotype, Type locality: leg. Erőss, Fehér, Grego, Szekeres, 28.06.2016 (HNHM 100167). Paratypes**,** same data (HNHM 100168/6; NHMW 111656/4; coll. Glöer 1 specimen and coll. Grego 5 specimens).

**Measurements.** H 1.7 mm; W 0.8 mm; WB 0.65 mm; HA/H 0.36, HB/H 0.26 (holotype). Paratypes have broken apices, measurements not provided.

**Etymology.** Derived from the name of Prekal village, where the type locality is situated.

**Description.** The whitish and silky shell has 5 tumid convex whorls with a deep suture and a blunt apex. The surface is smooth and shiny. The shell is elongated, slightly conical, and almost subcylindrical. Umbilicus is slit-like. In frontal view, the palatal side of the aperture protrudes laterally. Aperture is ovoid, slightly attached to the body whorl and slightly extended left of the columella line, its major diameter deviates by ca. 30 degrees from the plane of the columella. The peristome margin is sharp, slightly reflexed outwards. The evenly thick outer lip is sinuated and the lower part of the aperture protrudes forward in such a way, that in the lateral view the labral margin deviates ca. 13 degrees from the plane of the columellar axis.

**Habitat.** The Zhyla Cave is a prominent spring cave acting as a temporary water overflow channel during high water outlets in the main spring (Burimi i Zhylës) situated underneath the cave close to bank of Kiri River. The approximately 70 m long cave is formed in a dark grey limestone by a single phreatic tunnel of 10 × 5 meters average diameter and ends in a terminal siphon. The underground water stream is acoustically detectable under the large boulders close to the terminal siphon. The cave is flushed by the frequent water outflows leaving only fine sandy sediment on the bottom. Empty and fragmented shells were found inside the cave, in a large sand sediment deposits close to the terminal siphon at the end of the hitherto known main dry cave passage. Therefore, it is supposed to live deeper in the cave as a typical underground species colonizing also the appropriate water caverns and cave habitats.

**Distribution.** Only known from the type locality.

**Remarks.** Most of the collected specimens were empty shells of white color, but it is likely that the shells of living specimens have translucent yellowish color like all other related species known from live-collected material.

***Paladilhiopsis lozeki* sp. n.**

http://zoobank.org/796A594D-A35A-4655-8B61-FE461F31160D

Figs 5–6

**Diagnosis.** Differs from *Paladilhiopsis szekeresi* sp. n. (Albania, Tamarë) and *P. prekalensis* sp. n. (Albania, Prekal) by its larger and more elongated shell, less tumid more flattened whorls, its more acuminate apex, its narrower umbilicus and the shape of the aperture. *P. wohlberedti* sp. n. (Albania, Tamarë) has a significantly more conical shape with fewer whorls and different proportions as well as its larger, differently shaped aperture. *Montenegrospeum bogici* (Montenegro, Podgorica) from family Hydrobiidae has a larger conical shell with more tumid whorls and proportionally larger aperture. *Bosnidilhia vreloana* (Bosnia, Banja Luka) has a similar shell shape with fewer whorls, more tumid apical whorls, blunter apex, and a proportionally smaller, more elongated aperture.

**Type locality.** Albania, Tropojë district, Shoshan, Burimi i Shoshanit (Shoshan Spring), 270 m, 42.3862°N; 20.0795°E (Fig. 2B).

**Type material.** Holotype, Type locality, leg. Erőss, Fehér, Grego, Szekeres, 01.07.2016 (HNHM 100169).

**Measurements.** H 2.00 mm; W 0.85 mm; WB 0.75mm; HA/H 0.30, HB/H 0.26 (Holotype).

**Etymology.** Named after the well renowned Czech malacologist and an ever-helpful friend, Vojen Ložek (Prague, Czech Republic), who contributed significantly to the knowledge of Recent and Pleistocene mollusc fauna of Europe.

**Description.** The whitish shell has 5 ½ convex, slightly flattened whorls with a smooth surface, a semi-deep suture and a blunt apex. The shell has slim, elongated, subconical shape with ovoid aperture and closed umbilicus. The left side of the aperture extends very slightly beyond the columellar axis, declining from it by ca. 25 degrees. Aperture is situated under the last whorl and is not prominent on the shell periphery outline. The slightly outward reflexed peristome is equally thick along its outline. The labral lip is sinuous with forward declining lower end by 13 degrees from the columellar axis. The aperture is detached from last whorl by a tiny gap at the suture.

**Habitat.** Shoshan Spring is situated in a natural limestone amphitheatre close to left bank of the Valbona River, and it forms a prominent spring-fed lake supplied by water from multiple debris outlets at its southern side and from the adjacent waterworks outlet at its eastern side. A small side spring can be found at the northern side of the path to the waterworks building. An old watermill is located at the spring lake outlet to the Valbona River. The new subterranean species likely inhabits the interstitial water of the underground gravel sediment layer. Empty shell of the new species were collected in the sandy sediment of spring water outlet at the left side of the broad spring zone south of the waterworks building.

**Distribution.** Only known from the type locality, where it was found together with a spring gastropod that most likely belongs to the genus *Graeconatolica.* The latter species could be described once representative material becomes available.

***Paladilhiopsis szekeresi* sp. n.**

http://zoobank.org/60C0D5CC-3825-4608-942C-B5D5A6B9271D

Figs 7–8

**Diagnosis.** Differs from *Paladilhiopsis lozeki* sp. n. (Albania, Shosan) by its smaller and less elongate shell, more tumid whorls, less conspicuous apex, broader umbilicus, and the shape of the aperture. From *Paladilhiopsis prekalensis* sp. n. (Albania, Prekal) it differs by a more prominent apex, less convex whorls, less elongated and less declined aperture from the columella and by the broader umbilicus. The sympatric *Paladilhiopsis wohlberedti* sp. n. has a much more conical shell. *Montenegrospeum bogici* (Montenegro, Podgorica) from family Hydrobiidae has a slightly larger, more conical shell with more tumid whorls and proportionally larger aperture. *Bosnidilhia vreloana* (Bosnia, Banja Luka) has a somewhat similar shell with fewer, more tumid whorls, blunter apex, and a smaller, more elongate, oval aperture.

**Type locality.** Albania, Malësia district, Tamarë, a spring above the trout farm at the right side of valley, north of the village, 360 m, 42.4745°N; 19.5693°E (Fig. 2C).

**Type material.** Holotype: Type locality, leg. Erőss, Fehér, Grego, Szekeres, and 27.06.2016 (HNHM 100170), Paratypes: same data (NHMW 111657/1, further 1 specimen in coll. Glöer and 1 specimen in coll. Grego).

**Measurements.** Holotype H 1.7 mm; W 0.8 mm; WB 0.65 mm; HA/H 0.34; HB/H 0.28; Paratypes H: 1.5–1.8 mm, W: 0.7–0.9 mm. WB: 0.6–0.7 mm. Paratypes have broken apices, measurements not provided.

**Etymology.** Named after our colleague and friend, a prominent specialist of the family Clausiliidae, Miklós Szekeres (Szeged, Hungary), who accompanied us on the field trip in 2016

**Description.** The shell is whitish, consisting of 5, slightly convex whorls with a semi deep suture and a blunt rounded apex. The rapidly expanding first apical whorl makes the shell shape oval-elongate. The aperture is ovoid expanded on its right side, and the umbilicus is semi-opened and deep. The left side of the aperture is situated slightly left of the columellar axis. The upper part of the aperture is slightly detached from the body whorl at the suture.

**Habitat.** Empty shells of the new species were collected at sand among stones and inside a small concrete well-built directly on the spring zone. The only known locality is a permanent spring that arises at the junction of a large debris zone and the limestone massif (concrete well) with a second permanent outflow from a large debris about 30 m upstream the small valley. The lower active spring and well are connected to a pipeline and an aqueduct supplying a trout farm on the right bank of the Cem (Cijevna) River. A large temporary spring outlet is situated under a vertical limestone wall at the end of the deeply cut gorge about 100 m northwest of the two permanent springs, and the large oval boulders indicate a significantly higher temporary water flow during wet seasons and during the snow melting. The morphology of the gorge indicates its genesis by the collapse of a cave portal. The new species likely inhabits the interstitial water of the debris and the flooded caverns within a cave system present behind the debris.

**Distribution.** Only known from the type locality, where it was found syntopticaly with the more abundant *Paladilhiopsis wohlberedti* sp. n.

***Paladilhiopsis wohlberedti* sp. n.**

http://zoobank.org/8D8EA590-FE46-4BE7-AB2F-7BCAC0B85F3B

Figs 13–14

**Diagnosis.** *Paladilhiopsis wohlberedti* sp. n. differs from *P. prekalensis* sp. n. (Albania, Prekal), *P.lozeki* sp. n. (Albania, Shoshan) and *P. szekeresi* sp. n. (sympatric) by its more conical shell and proportinonately much larger body whorl. The new species can be compared by shell shape to *Paladilhiopsis grobbeni* Kuščer, 1928 (Slovenia, Sevnica), from which it differs by a more robust shell, more tumid whorls, blunter apex and by its differently positioned and shaped aperture. From *Paladilhiopsis szarowskae* it differs by its more conical shape, less (4 ½) and more tumid whorls, the more prominent body whorl and the larger aperture. *Montenegrospeum bogici* (Montenegro, Podgorica) has a more slender shell with more prominent and proportionally larger aperture situated more to the left of the columellar axis compared to the new species.

**Type locality.** Albania, Malësia district, Tamarë, a spring above the trout farm at the right side of valley, north of the village, 360 m, 42.4745°N; 19.5693°E (Fig. 1C).

**Type material.** Holotype: Type locality, leg. Erőss, Fehér, Grego, Szekeres, 27.06.2016 (HNHM 100171), Paratypes: same data (NHMW 111658/5; HNHM 100172/5; further 1 specimen in coll. Glöer and 14 specimens in coll. Grego).

**Measurements.** H 2.0 mm; W 1.0 mm; WB 0.9 mm; HA/H- 0.39, HB/H 0.33 (holotype).

**Etymology.** Named after Otto Wohlberedt (Triebes, Thüringen), who within his large work (1909) about Montenegro and northern Albania Mollusca (and Isopoda, Chilopoda, Diplopoda) contributed much to the knowledge of Invertebrate fauna of the Balkan peninsula.

**Description.** The whitish smooth shell has 4 ½ slightly tumid whorls with a semi-deep suture and a blunt apex. The shell shape is elongate-conoid with proportionally large body whorl. The oval aperture does not deviate from the conoid shell peripheral outline and is reflected, attached to the body whorl by its upper left side and extends slightly over the columellar axis on ts left side. The umbilicus is narrow and deep. On the lateral view, the labral peristome is straight and deviates backward by ca 8 degrees by the plane of columella.

**Habitat.** See *P. szekeresi* sp. n.

**Distribution.** Only known from the type locality, where it occurs syntopicaly with *P. szekeresi* sp. n.

***Paladilhiopsis falniowskii* sp. n.**

http://zoobank.org/C65B4812-FEBA-4BF0-B70C-882B5E8BC7A0

Figs 9–10

**Diagnosis.** Easily distinguished from the sympatric *Paladilhiopsis szarowskae* (Fig 12) by its larger and more oval shell, the more tumid and proportionally larger body whorl and the aperture shape. Compared to *Paladilhiopsis gittenbergeri*, (Albania, Vau i Dejes) (Fig 11) this species has slightly larger shell with a less prominent apex, less conical shape, and a differently shaped body whorl and aperture. *Paladihiopsis haduophylax*, Schütt, 1959 (Bileća, Hercegovina) shows some similarity, but its shells size is significantly larger, and the position and shape of the aperture is significantly different. *Paladilhiopsis blihensis* from Donji Kamengrad, Bosnia, has more slender shell with more (5) whorls, more acuminate apex and a different aperture shape. The *Iglica kanalitensis* A. Reischütz, N. Steiner-Reischütz & P.L. Reischütz, 2016, differs from the new species by its much more slender shape, smaller aperture and its aperture axis being less declined from the columella.

**Type locality.** Albania, Has district, Krumë, Vrela Spring, 450 m, 42.1921°N; 20.4166°E (Fig. 2D)

**Type material.** Holotype: Type locality, leg. Erőss, Fehér, Grego, Szekeres, 30.06.2016 (HNHM 100173), Paratypes: same data (NHMW 111662/1, one specimen in coll. Grego, two alcohol preserved specimens were destroyed during ongoing molecular studies at Jagellonian University).

**Measurements.** Holotype, H 2.4 mm; W 1.0 mm; WB 1.0 mm; HA/H 0.37; HB/H 0.35. Paratypes H: 2.3–2.4 mm, W: 0.9–1.0 mm, WB: 1.0–1.1 mm

**Etymology.** Named after the honored malacologist and friend, Andrzej Falniowski (Jagellonian University, Kraków), who has greatly contributed to the knowledge of the Balkan Hydrobiidae and Moitessieriidae.

**Description.** The white translucent smooth shell has 4 convex whorls with a semi-deep suture. The shell is elongate-ovoid, and the tumid whorls are regularly tapering towards the blunt apex. The aperture is ovate with very slightly reflexed margins, its palatal side does not exceed the shell periphery, and its columellar side extends beyond the columellar axis. The peristome is detached from the body whorl by, the umbilicus is a tiny furrow. The labrum is straight-not sinuated laterally

**Habitat.** The type locality is a karstic spring at the foot of a scree slope. It has a permanent outflow from within the right side of a shallow pond and an intermittent upper spring outflow, situated at about 5 m higher level, 50 m from active spring. The new species had been collected in the sandy sediment of the spring water outlet at bottom of the broad and shallow spring zone. There is a large communal waste deposit site near the spring zone, which poses a direct threat to the spring habitat and the fauna of the outflowing rivulet.

**Distribution.** Only known from the type locality. Within the type locality the new species co-occur with the subterranean *Plagigeyeria steffeki* sp. n., *Paladilhiopsis szarowskae* and several spring surface dwelling species: *Radomaniola curta* (Küster, 1852), *Ancylus fluviatilis* O.F. Müller, 1774, *Pseudamnicola krumensis* Glöer, Grego, Erőss and Fehér, 2015.

**Genus *Iglica* A. J. Wagner, 1910**

***Iglica illyrica*Schütt, 1975**

*Iglica* (*Rhaphica*) *illyrica* Schütt, 1975: 8, figs 26–27.

*Iglica illyrica* – Glöer et al. 2015: 71, fig. 3.

**Material.** Kosovo, Gjakova (Đakovica) district, Deçan (Dečani), Deçani luginë valley (dolina Dečani) 2 km NW behind the Visoki Dečani Monastery, along the left bank of Lumbardh i Deçanit (Dečanska Bistrica), W of the trout farm. 710 m, 42.5533°N; 20.2492°E; leg. Angyal, Erőss, Fehér, Grego, 25.6.2014

**Remarks.** This species is known only from the three locations, namely the spring of the White Drin River in Kosovo (Burimi i Drinit të Bardhë or Beli Drin Izvor, Fig. 2E), which is the type locality, from Kosovska Mitrovica in Kosovo and from Soljani near Rožaj in Montenegro (Schütt 1975). This new record extends its distribution range southwards along the foothills of Prokletije Mountains. We have to note, that during our visit in 2016, we observed significant disturbance of the spring habitat compared to 2014, when we first found this species. The extraordinary floods of near rivulet Lumbardh i Deçanit removed a large part of the left bank stone debris under the spring resulting in leaking the spring waters directly to the rivulet sediment and almost drying out the spring. As a result, no specimen of the species was found in the locality during our 2016 visit. We believe that the species is still present inside the underground waters and in the nearby waterworks installation.

**Family Hydrobiidae Stimpson, 1865**

**Genus *Plagigeyeria* Tomlin, 1930**

***Plagigeyeria steffeki* sp. n.**

http://zoobank.org/98763707-6C77-4400-B1FD-CE58FA321BA0

Figs 15–17

*Plagigeyeria gladilini* – Glöer et al. 2015: 80, figs 32–35.

**Diagnosis.** Compared to the most closely related *Plagigeyeria gladilini* Kuščer, 1937 (Kosovo, Novosellë) (Figs 18–20), the new species differs by its more slender shape, blunter apex, less prominent protoconch and narrower umbilicus as well as by the broader and more reflexed peristome with a wing-like columellar expansion. The lateral shape of the outer lip has a different sinuation in the two species. From *Plagigeyeria procerula* Angelov, 1965 (Bulgaria, Opizvet), it differs by its much larger shell and blunter apex as well as by the much wider and differently shaped reflexed peristome.

**Type locality.** Albania, Has district, Krumë, Vrela Spring, 450 m, 42.1921°N; 20.4166°E (Fig. 2D)

**Type material.** Holotype: Type locality: leg. Erőss, Fehér, Grego, Szekeres, 20.06.2016 (HNHM 100174), Paratypes: same data (NHMW 111659/40, HNHM 100175/62. Also 1 specimens in coll. Glöer and 69 specimens in coll. Grego).

**Other material.** Same locality, leg. Erőss, Fehér, Grego, 02.07.2015 (coll. Grego and coll. Eross).

**Measurements.** Holotype H 2.8 mm; W 1.7mm; WB 1.1 mm; HA/H 0.47, HB/H 0.28. Paratypes H: 2.2–3.1 mm, W: 1.5–1.9 mm., WB: 1.0–1.2 mm.

**Etymology.** Named after our untimely deceased excellent friend Jozef Šteffek (Banská Štiavnica, Slovakia) whose multidisciplinary activities dominated in the fields of malacozoology, ecosozology and zoogeography of the Slovakian mollusc fauna.

**Description.** The milky whitish, translucent shell has 4 convex whorls with a deep suture. The surface is smooth with fine axial growth lines. The shell is narrow-conical. The aperture is oval; the peristome expands widely outwards forming a wing-like structure at the columellar peristome. The lateral edge of the labrum is sinuated, and a characteristic sinuation is present on the wing shaped lower half of its columellar margin. The umbilicus is tiny, hidden behind the aperture and its reflected margins.

**Habitat.** See *P. falniowskii* sp. n.

**Distribution.** Only known from the type locality. Within the type locality the new species was found together with the subterranean *Paladilhiopsis falniowskii* sp. n. and *Paladilhiopsis szarowskae*.

**Remarks.** The first specimens of the new species were collected in 2015 and were erroneously reported as *P. gladilini* (Glöer et al. 2015). Since then, we collected further individuals from the Krumë population and obtained topotypical *P. gladilini* material at the White Drin Spring (Burimi i Drinit të Bardhë or Beli Drin Izvor, Fig 2E). Having these samples in hand made us conclude that *P. steffeki* is a distinct species, quite separable from *P. gladilini*.

*Plagigeyeria steffeki* sp. n. together with *P. gladilini* from Kosovo (Kuščer 1937) and *P. procerula* from Bulgaria (Angelov 1965), belong to a morphologically distinct group within the genus *Plagigeyeria* which is also geographically isolated from the other species of the genus. It would not be surprising if further molecular studies confirmed their separation at the genus level.

***Plagigeyeria zetaprotogona*Schütt, 1960**

Figs 21–28

*Plagigeyeria zetaprotogona* Schütt, 1960: 148–149, fig. 3 (Zetaquelle bei Tunjevo)

*Plagigeyeria zetadidyma* Schütt, 1960: 149–150, fig. 4 (Zetaquelle bei Tunjevo).

*Plagigeyeria zetatridyma* Schütt, 1960: 150, fig. 5 (Zetaquelle bei Tunjevo)

*Plagigeyeria montenigrina* Bole, 1961b: 207, fig. 3 (Obodska pećina bei Rijeka Crnojevića)

*? Plagigeyeria pageti* Schütt, 1961: 134–136, fig. 3 (Velika Spilja bei Risan)

*? Plagigeyeria pageti minor* Schütt, 1961: 136, fig. 3 (Velika Spilja bei Risan)

*Plagigeyeria zetaprotogona vitoja* Reischütz & Reischütz, 2008: 143, fig. 1 (Östliche Quelle in Vitoja am Ufer des Skadarsko Jezero):

*Plagigeyeria zetaprotogona zetaprotogona –* Schütt 1972: 115, fig.11.

*Plagigeyeria zetaprotogona zetadidyma –* Schütt 1972: 115, figs 12–13.

*Plagigeyeria zetaprotogona zetatridima –* Schütt 1972: 116, figs 14–16.

*? Plagigeyeria zetaprotogona pageti –* Schütt 1972: 116, figs 17–20.

*Plagigeyeria zetaprotogona montenigrina –* Schütt 1972: 116, fig.10.

*Plagigeyeria montenegrina* (*sic!*) – Radoman 1983: 107–108, figs 122–124.

*Plagigeyeria montenigrina* – Bank 2013

*Plagigeyeria zetaprotogona* – Bank 2013

*? Plagigeyeria zetaprotogona pageti* – Bank 2013

*Plagigeyeria zetaprotogona vitoja* – Bank 2013

*Plagigeyeria zetaprotogona zetadidyma* – Bank 2013

*Plagigeyeria zetaprotogona zetatridyma* – Bank 2013

*Plagigeyeria zetaprotogona* – Glöer et al. 2015: 74, figs 7–8.

**Material.** Albania, Malësia district, Bajzë, Syri i Sheganit Spring by the Shkodër Lake, 10 m, 42.2722°N; 19.3963°E (Fig.2F); leg. Erőss, Fehér, Grego, Szekeres, 27.06.2016 (coll. Grego, HNHM 100385, NHMW 111661) – Shkodër district, Rrash, Burimi te Vrakut (Kroi i Vrakes, Vrak Spring), 60 m, 42.1457°N; 19.5452°E; leg. Erőss, Fehér, Grego, Szekeres, 28.06.2016 (coll. Grego) – Malësia district, Shpellë e Dverptes (Dverpte Cave), 4.5 km S of Tamarë, 200 m, 42.4262°N; 19.5272°E; Erőss, Fehér, Grego, Szekeres, 27.06.2016 (coll. Grego) (Fig. 2G).

**Remarks.** The taxonomic identity of this species is not clear. Schütt (1972) associated five subspecies with it, while Radoman (1983) didn’t even mention this name is his monograph. In Schütt’s (1972) opinion the typical *montenigrina* can be considered the morph from which the others are derived. Bank (2013) mostly follows Schütt’s (1972) view, except that treating *montenigrina* a distinct species. The source of uncertainty is that, unlike in other subterranean (and epigean) truncatelloid snails, striking morphological differences could be observed within the same museum lots (found at the same localities), e.g. three different morphs were described from the Zeta Spring in Tunjevo. Schütt (1972) found that two or three morphotypes can often be detected in the same location. Based on the biological species concept, one could argue that co-existence of these morphs is a strong argument against their distinction at the subspecies level. However, it is also conceivable that the museum lots studied by Schütt (1972) represent thanatocoenoses only, i.e. they were washed together from various, geographically isolated subpopulations, and therefore the co-existence of the various morphs is just artifactual. This makes it questionable how many species and subspecies actually exist, and, if there is more than one species, how large is the geographic range of *Plagigeyeria zetaprotogona* s. str.

We have found three populations that seem to belong to this taxon (or taxon group), which are the first records thereof from Albania. The Syri i Sheganit population is conchologically very similar to that of the Vitoja Spring (Podgorica, Montenegro, type locality of *P. z. vitoja*), and the other two populations further expand the known range of morphological heterogeneity. The specimens from Vrak spring have a more robust shape and could represent a new subspecies, while the single broken shell and few fragments from Shpelle Dverptes are more elongate. Until more material become available we will treat it as *Plagigeyeria* cf. *zetaprotogona*.

**Genus *Vinodolia*Radoman, 1973**

Figs 29–32

***Vinodolia matjasici* (Bole, 1961)**

*Iglica matjašiči* Bole, 1961a: 59–60, fig. 1 (a small spring near Rijeka Crnojevića)

*Anagasta matjašiči* – Radoman 1973: 423, fig. 2C.

*Iglica* (*Iglica*) *matjasici –* Schütt 1975: 7, figs 21–22.

*Anagastina matjasici* – Radoman 1983: 54–55, fig. 45.

*Vinodolia* (*Anagastina*) *matjasici* – Bank 2013

*Vinodolia matjasici* – Pešić and Glöer 2013a: 79.

*Vinodolia matjasici* – Glöer et al. 2015: 74, fig. 9

**Material.** Albania, Malësia district, Bajzë, Syri i Sheganit Spring by the Shkodër Lake, 10 m, 42.2722°N; 19.3963°E (Fig.1F), leg. Erőss, Fehér, Grego & Szekeres, 27.06.2016 (coll. Grego).

**Remarks.** Hitherto only three locations of this species were known, all in Montenegro: the type locality near Rijeka Crnojevića (Bole 1961a), more precisely Lipovik Spring near Rijeka Crnojevića (according to Radoman 1973, 1983); Vilina Cave near Virpazar (Schütt 1975) and Vitoja Spring (Glöer et al. 2015). This new record confirms the presence of this species in Albania and extends its known distribution within the Skadar Lake drainage. All known populations show some difference in the shell size and morphology, but the scarce material does not allow us to understand their significance, and thus, for the time being, we consider the above mentioned populations conspecific.

**Genus *Saxurinator*Schütt, 1960**

***Saxurinator schlickumi*Schütt, 1960**

Figs 27–28

*Saxurinator schlickumi* Schütt, 1960: 148, fig. 2 (Rugovska klisura bei Peć).

*Saxurinator schlickumi* – Bank 2013

**Material.** Kosovo, Pejë (Peć) district, Rugova Gorge, 3.5 km W of the Patriarchate Monastery, spring with travertine waterfalls at the left side of gorge near a resting station, 650 m, 42.6654°N; 20.2307°E; leg. Erőss, Fehér, Grego, Szekeres, 03.07.2016 (NHMW 111660, HNHM 100384)

**Remarks.** This species was known only from the type locality, which was defined quite inaccurately as ”Rugovska klisura bei Peć” (=Rugova Gorge or Gryka e Rugovës, near Pejë) by Schütt (1960). It has not been found since, and, as the Rugova Gorge is 25 kilometers long and there are numerous springs along both sides, it remained uncertain where the type material originated and where exactly does the species live. Due to the lack of any recent data to validate the species’ distribution, Seddon (2011) assessed it as Data Deficient (DD). The Rugova Gorge is a protected site now, and there is no reason to suppose that there is any threat to its subterranean habitats. This record confirmed the presence of this species and makes us suppose that it might be found also in other springs within the gorge.

## Discussion

As before, little is known about how environmental factors influencing the living conditions of subterranean truncatelloid gastropods. Most of our knowledge is based on field observations and partly on intuition of the specific groundwater habitat characterization. We assume that the most important factors determining the appropriate habitat of the subterranean freshwater species include: *1.- the absence of daylight; 2.- the availability of food; 3.- oxygen and carbon dioxide concentrations of the water; 4.- water chemistry;* and *5.- water flow velocity.* Combinations of these factors within their optimal range for the snail species determine their living conditions directly and indirectly by influencing the composition of the biotic community (influence on the growth of food species, on predator species, and on the competitors, parasites, etc.) as well.

### 1 Absence of daylight

By the absence of light, photosynthesizing Cyanobacteria, algae and auxotrophic Labyrinthulomycetes are also absent in the subterranean habitats, while their strong presence on the surface spring zone has allowed the evolution of a wide range of daylight dependent food competitors, predators and parasites. We assume that the lower number of predators, parasites and competitors for food, together with the more steady environment was the main driving factor for gastropods inhabiting and adapting to underground habitats.

### 2 Availability of food

Subterranean truncatelloids are presumably feeding on the chemolithotrophic prokaryotes gaining the energy for their biochemical processes (structural proteins, lipids and sugar synthesis) under the absence of light from the oxidation of inorganic cations, in most cases Fe^2+^ or Mn^2+^. In similar some thermal and mineral waters (and deep sea vents) could host ecosystems based on biological oxidation of sulphurous compounds and H_2_S by chemoautothropic microorganisms (Falniowski at al. 2008). The organic material accumulated by the chemolithotroph/chemoautotroph assemblages is thus a fundament for specific underground ecosystems. The precipitated inorganic residua of chemolithotropic process (hydroxides/oxides of Fe^3+^ or Mn ^4+^ can be frequently found accumulated on the outer shell surface of underground gastropod species as a brown or black overlay indicating the likelihood of our assumption. We have also an indication that some underground valvatiform species (Carpathian populations of *Hauffenia*) can feed on the dense net of soft tiny tree roots penetrating the interstitial water among debris within the spring zone. Likely the flat valvatiform shell shape could better trap those species in the net of roots. Future genetic investigations of gastropod intestine content could most likely prove the above theories.

### 3 Oxygen and CO_2_ saturation

Dissolved oxygen is necessary for gastropod breathing and also to oxidative reactions important for the chemolitotrophic processes within its feed chain, while the dissolved CO_2_ is essential as the main carbon source for the feed bacteria. Both saturations are strongly dependent on temperature (saturation increasing by lower temperature) and thus the environment temperature has a secondary effect on optimal saturation range for the gastropod as well as food microorganisms. Furthermore, the CO_2_ saturation coupled with dissolved hydrocarbonate equilibrium are the key factors driving the corrosive/accumulation character of the karstic water. Some underground gastropod species are also adapted to thermal extremes and waters with high CO_2_, H_2_S content, and low oxygen saturation (Falniowski et al. 2008).

### 4 Water chemistry

The dissolved ions are important for reactions driving the biomass growth (Fe^2+^, Mn^2+^) of the food species. The calcium content is important to the secretion of the gastropod shell and other water-soluble growth factors are essential for the gastropod as well for the food substrate, in addition it stabilizes the pH value (hydrocarbonate equilibrium). As all the underground species are narrowly specialized to their steady chemical habitat, even slight anthropogenic pollution or other environment alternation could have a severe impact on water chemistry and could cause irreversible damages on the whole underground ecosystem.

### 5 Water flow velocity

The water flow velocity influences the oxygen and CO_2_ saturation and distribution. We assume it is also important for the species motility within the habitat. Stagnant water has lower oxygen saturation and higher CO_2_ content coming from decaying organic material. Low flow allows better animal motility towards food and to sexual partners and the opposite for high. High flow also challenges the adhesive strength of gastropod musculature and slime important for attachment of the animal to the substrate. The seasonal very high water flow likely detaches live animals from the substrate and washes them out where they accumulate in the sedimentation zones. The underground species must have adapted to withstand the flowing water within their habitat. We are convinced that the average water flow velocity could also determine the shape of the shell: more slender shells can stand higher velocities of water due to lower hydrodynamic resistance and the more robust forms evolved in habitats with slower or steady water velocity.

Although they are often referred to as cave-dwellers, the subterranean truncatelloids (those discussed in this paper and *Bythiospeum*, *Paladilhia*, *Hauffenia*, *Islamia*, *Lanzaia* etc.) are not restricted only to the caves and cave waters. Based on our two decades of field experience from the Carpathian mountains, the Carpathian basin, Dinarid Alps and southeastern Asia (Šteffek and Grego 2002, 2005; Šteffek et al. 2011; Glöer and Grego 2015; Glöer et al. 2015; Erőss and Petró 2008; Pešić and Glöer 2012, 2013a, 2013b; Glöer and Pešić 2014a, 2014b), we can conclude that their habitat is the groundwater saturated zone, including interstitial spaces within the coarser sand and gravel deposits, among debris as well as inside the crevices and caverns of the bedrock. On limestone substrate they might also occur in larger cavities and caves as well, and these, together with spring outlets and wells, are the usual places where they can be sampled relatively easily.

Some facultative cave-dwelling species (Šteffek and Grego 2005) can mainly be found in the spring zone and spring debris, while their occurrence inside the cave waters is restricted to the passages close to the spring zone and they their occurrence is not extended into the deeper parts of the cave systems. Several taxa/local populations, occurring in non-limestone habitats with optimal water chemistry (e.g. *Hauffenia kissdalmae* Erőss & Petró, 2008 in the volcanic Börzsöny Mts., Hungary; *Bythiospeum oshanovae* (Pintér, 1968) in the gravel of the Danube, Hungary; *Hauffenia* sp. in sandstones of the Rimavská Basin, Slovakia; *Alzoniella slovenica* (Ložek & Brtek, 1964) in the outer Carpathian flysh, Slovakia and Czech Republic, *Bythiospeum carpathicum* (Soós, 1940) on the flysh of Mt. Hoverla, Ukraine) indicate that subterranean truncatelloids are not bound exclusively to karstic systems.

Considering the above discussed very specific range of habitats the discussed gastropod species cannot be called as “troglobiont” species, as this term is preferably used for mostly air breathing species inhabiting the cave areas above the water level. The term “freshwater troglobiont” suggested by Sket (2008) also does not fit to the above described habitat range, as the species are not limited to cave waters. The term “stygobiont” is also not well defined and very differently interpreted, most frequently used to describe the relevant habitat (Bole and Velkovrh 1986). With this respect we would prefer the interim term “subterranean freshwater habitat” which better covers the broad habitat ranges and makes the understanding of the subject clearer, until some other generally accepted and more appropriate term is adopted.

## Conclusions

While the neighboring former Yugoslavian territories have been recognized for their large subterranean freshwater gastropod species diversity for a long time, no data had been reported from Albania before the end of the millennium (Radoman 1983, 1985; Bole and Velkovrh 1986; Kabat and Herhsler 1993). As several other molluscan groups, terrestrial gastropods among others, showed a similar pattern in the Balkans, the question arose as to whether Albania is actually a gap in continuation of the Balkan diversity or just *terra incognita* (Sattmann and Reischütz 1994). The malaco-faunistic exploration in Albania gathered momentum only after the political transition of the country in 1990 (Welter-Schultes 1996, Fehér et al. 2004), and this resulted in the discovery of the first subterranean freshwater gastropod population only in 2006. This was first reported as *Paladilhiopsis* cf. *serbica* (Pavlović, 1913) by Fehér and Erőss (2009), but considered later as a presumably distinct species (Reischütz et al. 2013). Since then, including the six new species reported within this article, the number of subterranean freshwater species described from Albania has increased to 11 (Reischütz and Reischütz 2008; Reischütz et al. 2014, 2016, Glöer et al. 2015), and the total known Albanian fauna is now comprised of 16 species including the two new records herein and the 3 known undescribed species. During our field trip in 2016 we sampled 16 springs and cave outflows altogether, and, in fact, we discovered new species in ca. a quarter of them while underground species were detected in almost half of the investigated localities. This suggests that these known species represent just a small fraction of the actual species richness, and most of the Albanian subterranean gastropod diversity is still waiting to be discovered. We hope that this study will stimulate future research toward a better understanding of these cryptic ecosystems in Albania.

## Figures and Tables

**Figures 3–8:**
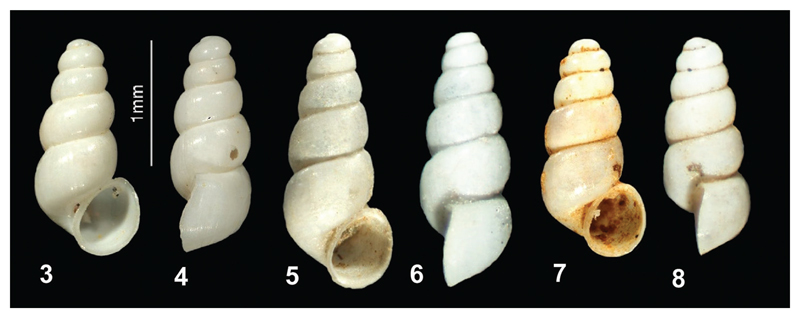
**3–4** *Paladilhiopsis prekalensis* sp. n. (holotype) **5–6** *Paladilhiopsis lozeki* sp. n. (holotype) **7–8** *Paladilhiopsis szekeresi* sp. n. (holotype); (photos: Peter Glöer, Jozef Grego).

**Figures 9–14:**
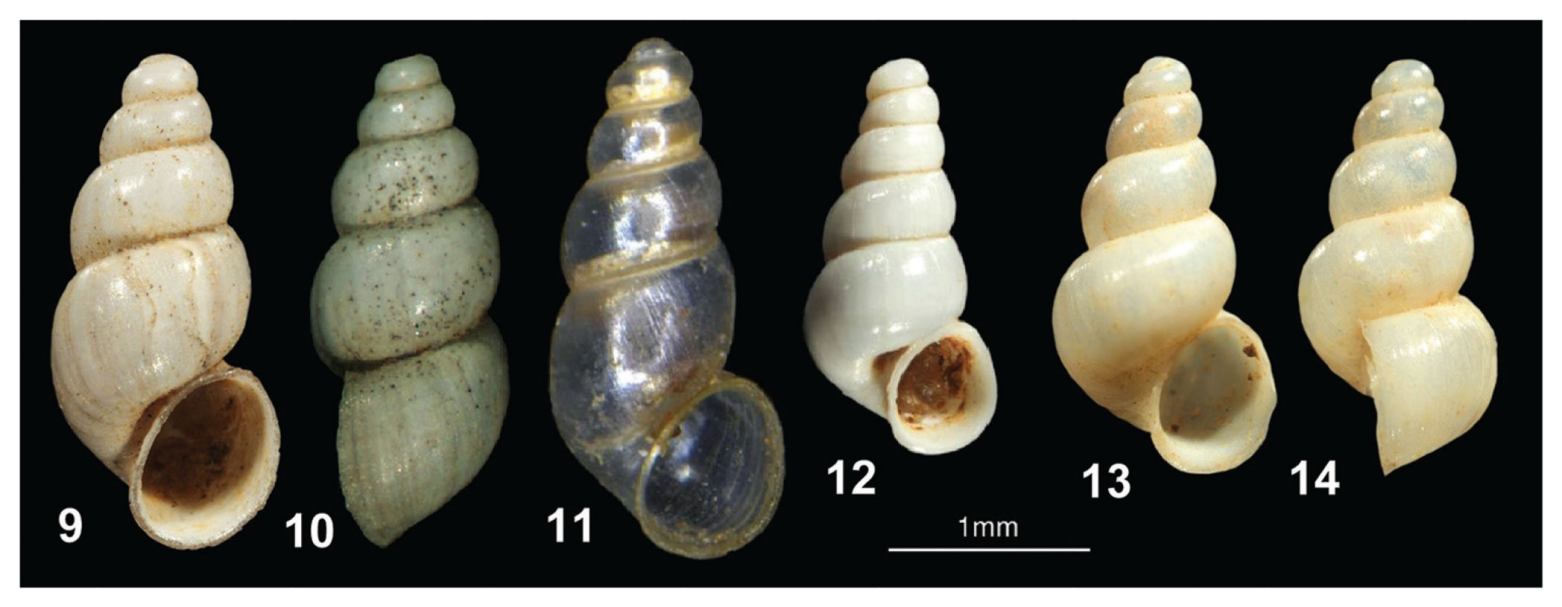
**9–10** *Paladilhiopsis falniowskii* sp. n. (holotype) **11** *Paladilhiopsis gittenbergeri* (holotype) **12** *Paladilhiopsis szarowskae* (holotype) **13–14** *Paladilhiopsis wohlberedti* sp. n. (holotype); (photos: Peter Glöer, Jozef Grego, Zoltán Fehér).

**Figures 15–20:**
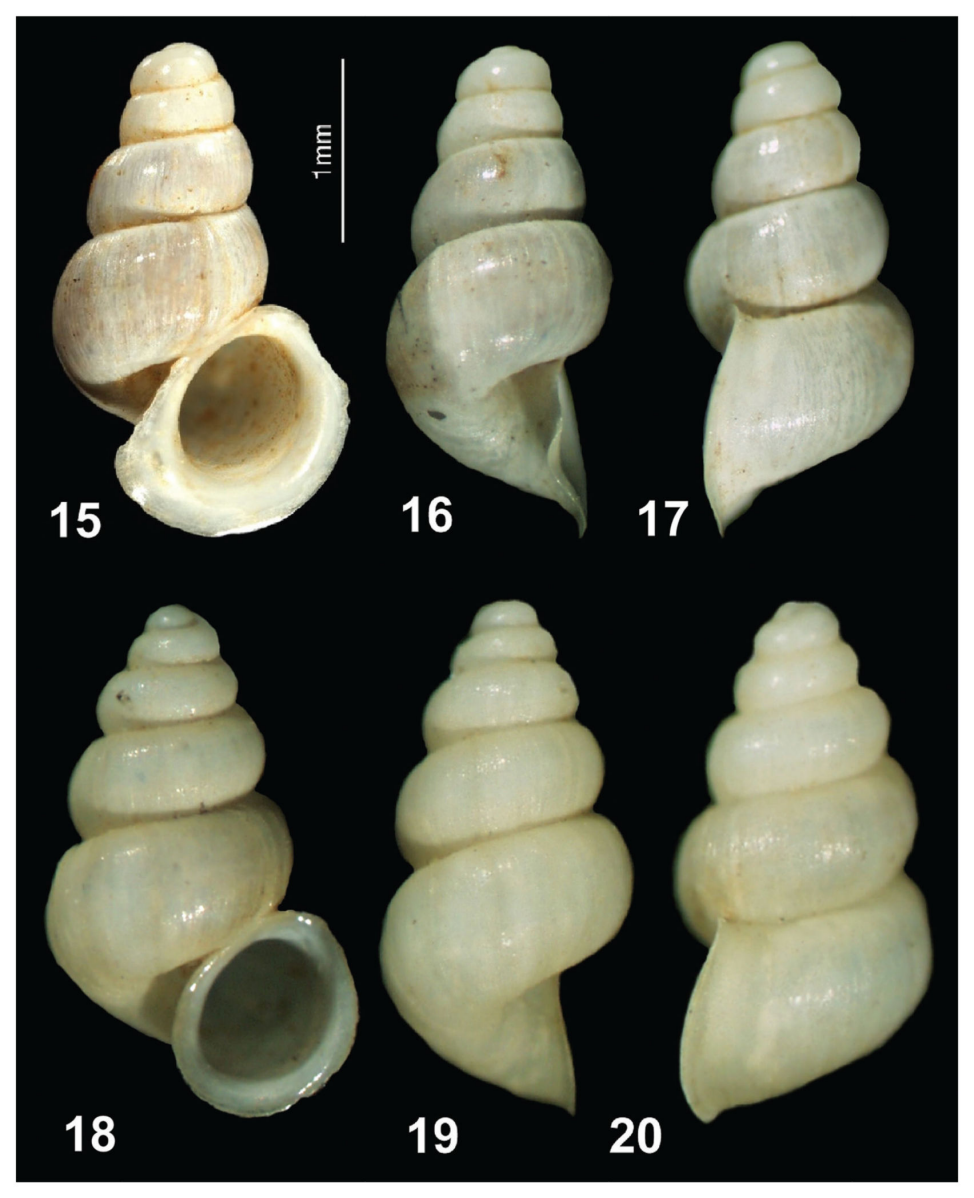
**15–17** *Plagigeyeria steffeki* sp. n. (holotype) **18–20** *Plagigeyeria gladilini* (topotype from the White Drin Spring near Novosellë).

**Figures 21–28:**
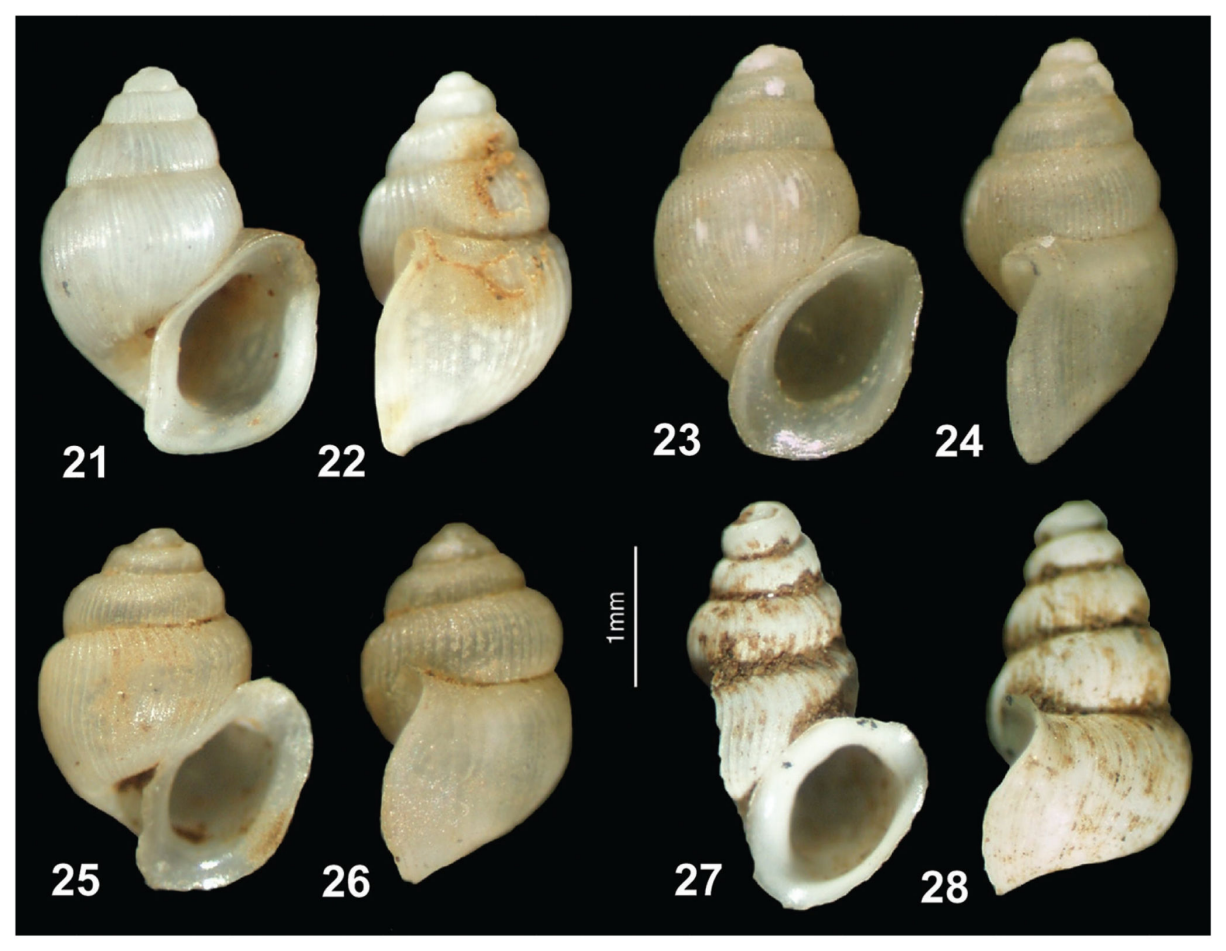
**21–26** *Plagigeyeria zetaprotogona*: **21–22** Montenegro, Vitoja Spring **23–24** Albania, Bajzë, Syri i Sheganit Spring **25–26** Albania, Rraps, Vrak Spring; *Plagigeyeria* cf. *zetaprotogona*: **27–28** Albania, Dverpte Cave. (photos: Peter Glöer, Jozef Grego).

**Figures 29–34:**
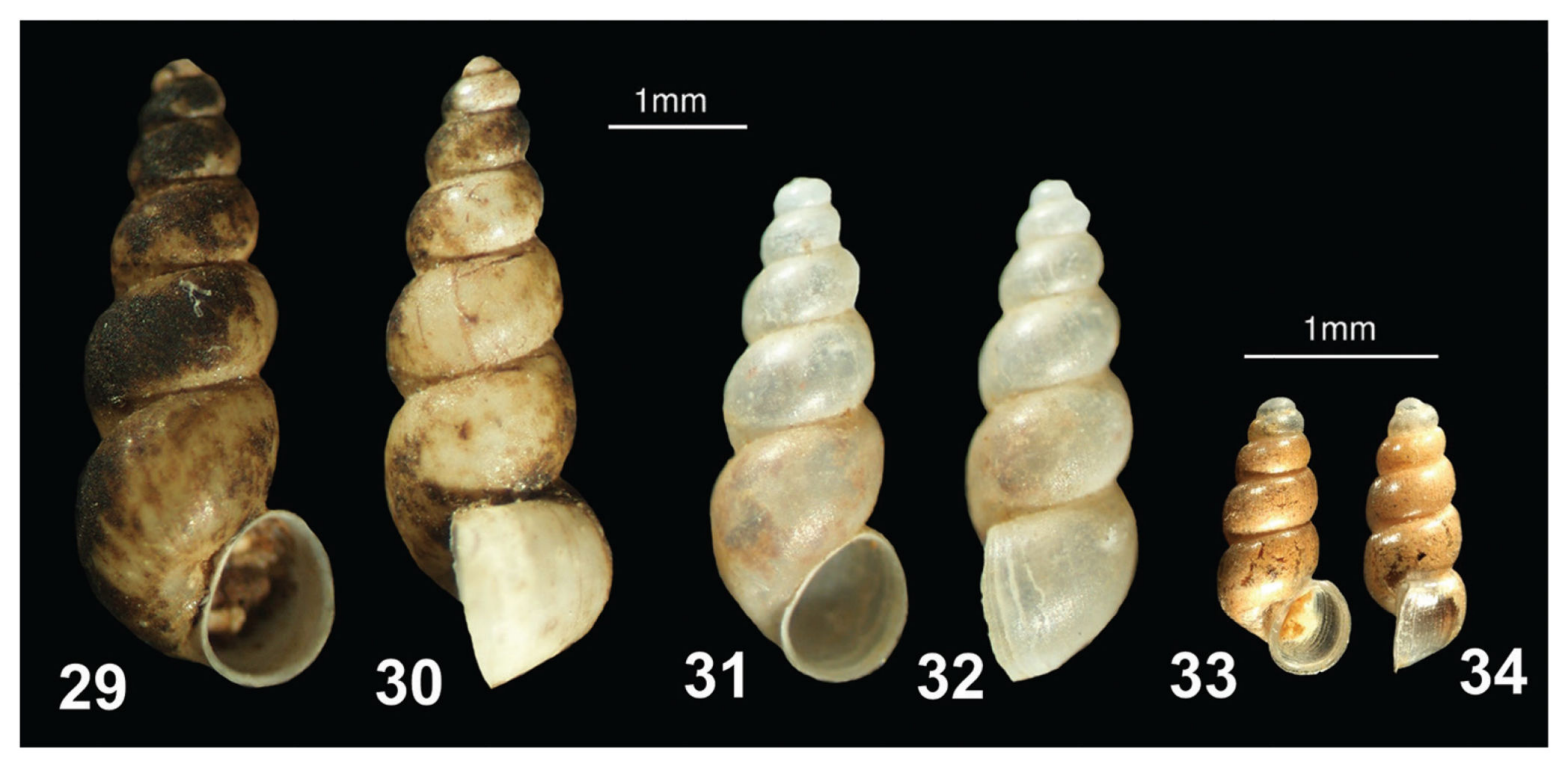
**29–32** *Vinodolia matjasici*: **29–30** Albania, Bajzë, Syri i Sheganit **31–32** Montenegro, spring Vitoja **33–34** *Saxurinator schlickumi*: Kosovo: Peja, Rugova Gorge; (photos: Peter Glöer, Jozef Grego).

## Abbreviations

HNHM: Hungarian Natural History Museum, Budapest
NHMW: Naturhistorisches Museum, Wien
H: Shell height
W: Shell width
WB: Width of the body whorl
HA: Aperture height
HB: Height of the body whorl
